# Multiomics analysis reveals the exacerbating effect of constipation on autism-related symptoms in children with autism spectrum disorder

**DOI:** 10.1038/s41522-025-00894-5

**Published:** 2026-01-08

**Authors:** Hailin Li, Xiuhong Li, Xin Wang, Lizi Lin, Muqing Cao, Shuolin Pan, Xiaoxuan Ou, Tingfeng Gu, Shuli Shen, Hailin Li, Jin Jing

**Affiliations:** 1 Department of Child and Adolescent Health and Health Education, School of Public Health, Inner Mongolia Medical University, Hohhot, China; 2 School of Public Health, Shenzhen Campus of Sun Yat-Sen University, Shenzhen, China; 3 Laboratory of Brain, Cognition and Education Sciences, Ministry of Education; Institute for Brain Research and Rehabilitation, and Guangdong Key Laboratory of Mental Health and Cognitive Science, South China Normal University, Guangzhou, China; 4 Department of Maternal and Child Health, School of Public Health, Sun Yat-Sen University, Guangzhou, China; 5 School of Sport and Health, Guangzhou Sport University, Guangzhou, China; 6 Department of Child Health, Guangzhou Women and Childrens Medical Center, Guangzhou, China; 7 Department of Emergency Management and Health Security, Guangzhou Center for Disease Control and Prevention (Guangzhou Health Supervision Institute), Guangzhou, China

**Keywords:** Microbiology, Health care

## Abstract

This study investigated the relationship between constipation and autism-related symptoms in children with autism spectrum disorder (ASD). Participants were assessed for gastrointestinal (GI) and autism-related symptoms and classified into constipated and non-constipated groups. The relationship was further explored via 16S rRNA sequencing and non-targeted metabolomics to identify underlying mechanisms. Results revealed that constipated ASD children exhibited more severe autism-related symptoms and alterations in four bacterial taxa—the phylum Bacteroidetes, the family Barnesiellaceae, and the genera *Alistipes* and *Bilophila*—plus 451 metabolites compared to non-constipated ASD children. Among the altered bacterial taxa, three—Bacteroidetes, *Alistipes*, and *Bilophila*—exacerbated the relationship between constipation and autism-related symptoms. Five metabolites derived from the above three taxa—chenodeoxycholic acid, palmitic acid, glutaric acid, arachidonic acid, and choline—were significantly associated with autism-related symptoms. Our multi-omics analysis reveals the exacerbating effect of constipation on autism-related symptoms in children with ASD.

## Introduction

Numerous studies have consistently revealed that children with autism spectrum disorder (ASD) exhibited more frequent and severe gastrointestinal (GI) symptoms compared to typically developing (TD) children^1,2^. Multicenter studies in 13 cities of China demonstrated that the detection rates of constipation (40.1% vs 25.6%), stool odor (17.0% vs 9.3%), and overall GI symptoms (53.6% vs 41.3%) were markedly higher in children with ASD compared to those in TD children, suggesting potential geographical pervasiveness of autism-related GI dysfunction^3^. Among the spectrum of GI symptoms associated with ASD, constipation emerges as one of the most frequently reported and clinically significant^4^. Nevertheless, the potential association between constipation and the core symptomatology of ASD has been largely underexplored. Moreover, despite robust clinical evidence and consistent parental reports underscoring the detrimental impact of constipation on the quality of life, social functioning, and emotional regulation in children with ASD^5,6^, there remains a paucity of rigorous, in-depth studies specifically investigating this correlation. Elucidating the relationship between constipation and autism-related symptoms is critical to advancing our understanding of their potential interplay and its implications for neurodevelopmental outcomes.

Studies have shown that food selectivity issues in children with ASD reduce the microbial diversity in the gut^7^. This decreased diversity is hypothesized to contribute to GI symptoms such as abdominal pain and irregular stool consistency, ranging from loose to hard stools^7^. Research by Kang et al. elucidated that autism with co-occurring GI symptoms exhibits a distinct gut bacterial taxa profile characterized by reduced microbial diversity, specifically featuring lower abundances of *Prevotella*, *Coprococcus*, and unclassified Veillonellaceae^8^. Other research has found higher levels of Bacteroides vulgatus and Desulfovibrio species in ASD children with GI symptoms compared to controls, where Firmicutes were typically more predominant in control subjects (7 non-autistic siblings and 8 control subjects)^9^. Additionally, Tomova et al. observed a very strong association of the amount of *Desulfovibrio* spp. with the severity of GI dysfunction and autism symptoms^10^. While correlation does not equate to causation, the available evidence suggests children with autism who experience more severe GI symptoms also tend to have higher autism severity scores^11^. This correlation may be influenced by the reduced diversity and altered composition of the gut microbiome.

It has been proposed that the metabolites could cross the blood-brain barrier and affect receptors in the neurons, thus affecting the development and signaling of brain^12^. Therefore, alterations in gut microbiota composition, and in the metabolites produced by them, may contribute to or worsen autism-related symptoms^13^. Rose et al. suggested that children with ASD who experience GI symptoms have an imbalanced immune response pattern, possibly influenced by metagenomic and metabolomic changes, and may have a propensity to impaired gut barrier function, which may contribute to their symptoms and clinical outcome^14^. Zarimeidani et al. critically examined recent evidence and emphasized the potential role of neuroinflammatory intermediates that are linked to gut microbiota alterations in individuals with ASD^15^. Growing evidence implicates dysregulated neuroinflammation in the pathophysiology of ASD^16^. These findings underscore the intercommunication between gut microbiota, microbial metabolites, and neurodevelopmental impairments in children with ASD. Accordingly, we hypothesized that GI symptoms, particularly constipation, may be linked to autism-related symptoms, and that gut microbiota and their metabolites may serve as potential mechanistic moderators in these correlations.

In summary, while constipation is a prevalent GI symptom in children with ASD, its association with the core symptoms of ASD, as well as emotional and behavioral problems, remains poorly understood. This study aims to explore the specific correlation between constipation and autism-related symptoms, as well as its potential mechanisms involving gut microbiota and their metabolites. By addressing these critical gaps, our findings have the potential to pave the way for the development of targeted therapeutic strategies, ultimately enhancing clinical outcomes and improving the quality of life for these affected individuals.

## Results

### Demographic characteristics of the study subjects

The final participants comprised 90 children with ASD, stratified by constipation status (*n* = 30 with constipation; *n* = 60 without). Demographic characteristics are detailed in Table 1. The median age for children with ASD in the constipation group was 6.1 years (range, 5.0–7.0 years), with approximately 86.7% being boys. In the non-constipation group of ASD children, the median age was 6.1 years (range, 4.4–7.1 years), with about 80% being boys. The median diagnostic ages for the constipation group and the non-constipation group of ASD children were 2.8 and 3.5-years-old, respectively. Significant differences between the constipation and non-constipation groups of ASD children were observed only in age at diagnosis and maternal GI symptoms during pregnancy (*P-*value = 0.043, *P-*value = 0.034; effect size = 0.214, effect size = 0.223).

**Table 1:** Demographic characteristics of the ASD constipation group and the ASD non-constipation group (*n* = 90)

Characteristics	Constipation (*n* = 30)	Non-constipation (*n* = 60)	*P-*value	Effect size
**Child characteristics**				
Age, median (IQR), y	6.1 (5.0, 7.0)	6.1 (4.4, 7.1)	0.716	0.038
Sex, *n* (*n*% of males)	26 (86.7)	48 (80.0)	0.436	0.082
BMI, median (IQR), kg/m^2^	15.1 (14.4, 16.4)	15.4 (14.4, 16.6)	0.751	0.033
Age at diagnosis, median (IQR), y	2.8 (2.5, 3.7)	3.5 (2.6, 5.0)	0.043	0.214
Early intervention before the age of 5, *n* (%)	29 (96.7)	49 (81.7)	0.055	0.208
ASD symptom severity^a^, *n* (%)				
Mild-to-moderate	24 (80.0)	48 (80.0)	1.000	0.000
Severe	6 (20.0)	12 (20.0)		
Intellectual functioning^b^, *n* (%)				
Normal	12 (40.0)	26 (43.3)	0.727	0.062
Borderline	4 (13.3)	5 (8.3)		
Abnormal	14 (46.7)	29 (48.3)		
Premature birth, *n* (%)	4 (13.3)	2 (3.3)	0.093	0.189
Birth mode, *n* (%)				
Vaginal delivery	17 (56.7)	36 (60.0)	0.758	0.056
Elective cesarean section	5 (16.7)	12 (20.0)		
Emergency cesarean section	8 (26.7)	12 (20.0)		
Birth order, *n* (%)				
1	19 (63.3)	37 (61.7)	0.878	0.016
> 1	11 (36.7)	23 (38.3)		
Feeding patterns before 2 years of age^c^, *n* (%)				
Breastfeeding	4 (13.3)	19 (31.7)	0.139	0.148
Mixed feeding	7 (23.3)	14 (23.3)		
Artificial feeding	19 (63.3)	27 (45.0)		
Food allergy, *n* (%)	4 (13.3)	12 (20.0)	0.436	0.083
Antibiotic exposure before the age of 5^d^, *n* (%)	16 (53.3)	42 (70.0)	0.119	0.164
Sleep duration, median (IQR), h/day	9.6 (9.0, 10.4)	9.8 (9.2, 10.7)	0.390	0.091
SB, median (IQR), h/d	3.0 (2.0, 5.1)	2.5 (1.0, 4.1)	0.137	0.157
MVPA, median (IQR), h/d	0.6 (0.2, 1.1)	0.8 (0.4, 1.1)	0.288	0.112
Walking, median (IQR), h/d	0.5 (0.3, 1.0)	0.5 (0.2, 1.0)	0.345	0.100
**Maternal characteristics**				
Advanced maternal age, *n* (%)	0 (0.0)	5 (8.3)	0.165	0.172
Educational attainment, *n* (%)				
College degree or below	17 (56.7)	31 (51.7)	0.654	0.047
Undergraduate degree or higher	13 (43.3)	29 (48.3)		
Gestational diabetes mellitus, *n* (%)	7 (23.3)	7 (11.7)	0.216	0.152
Maternal obesity, *n* (%)	2 (6.7)	1 (1.7)	0.257	0.131
GI symptoms during pregnancy, *n* (%)	11 (36.7)	10 (16.7)	0.034	0.223
Antibiotic exposure during pregnancy, *n* (%)	0 (0.0)	3 (5.0)	0.548	0.131
**Family characteristics**				
Monthly per-capita income, *n* (%)				
≤ 8000 RMB	22 (73.3)	43 (71.7)	0.868	0.018
> 8000 RMB	8 (26.7)	17 (28.3)		
Parenting behavior^e^, *n* (%)				
Support/engagement	26 (86.7)	56 (93.3)	0.433	0.111
Opposition/defiance	4 (13.3)	4 (6.7)		

*ASD* autism spectrum disorder, *IQR* interquartile range, *BMI* body mass index, *SB* sedentary behavior, *MVPA* moderate-to-vigorous physical activity, *GI* gastrointestinal, *RMB* Ren Min Bi (Chinese currency).

^a^Evaluated by the childhood autism rating scale (CARS).

^b^Evaluated by the Wechsler Intelligence Scale for Children Fourth Edition (WISC-IV) or Gesell developmental Schedules (GDS).

^c^Longest sustained feeding patterns before 2 years of age.

^d^Children have taken antibiotics or antifungal medications before the age of 5 years.

^e^Evaluated by the Parent Behavior Inventory (PBI). Effect size = Z/√(N) for Mann–Whitney *U*-tests, and effect size φ or Cramer-V for *χ*^2^ tests.

### Comparisons of autism-related symptoms between the constipation and non-constipation groups of children with ASD

Univariate analysis revealed significantly higher scores for social awareness, social communication, and the social responsiveness scale, second edition (SRS-2), total scores in constipated ASD children compared to their non-constipated counterparts (all effect sizes > 0.1). Additionally, the scores for emotional symptoms and the strengths and difficulties questionnaire (SDQ) total scores in the ASD constipation group were significantly higher than in the non-constipation group of children with ASD (all effect sizes > 0.1), as illustrated in Table 2.

**Table 2:** Comparisons of autism-related symptoms between the constipation and non-constipation groups of children with ASD (*n* = 90)

Variables	Constipation (*n* = 30)	Non-constipation (*n* = 60)	*P*- value	Effect size
ASD symptom severity	28 (26.5, 35.75)	27.5 (26.5, 34.5)	0.664	0.046
Social deficits				
Social awareness, median (IQR)	12 (11, 13.25)	11 (9, 13)	0.048	0.209
Social cognition, median (IQR)	20 (15.75, 23)	18 (15, 22)	0.150	0.152
Social communication, median (IQR)	33.5 (28, 40.25)	30 (24, 36.75)	0.067	0.193
Social motivation, median (IQR)	14.5 (10.75, 20.25)	13.5 (10, 17)	0.278	0.114
Autistic mannerisms, median (IQR)	16.5 (10.75, 20)	13.5 (9, 19)	0.474	0.075
Total scores, median (IQR)	97 (83.25, 111.25)	88 (72.5, 105)	0.064	0.195
Emotional and behavioral problems				
Emotional symptoms, median (IQR)	3 (2, 4)	2 (1, 4)	0.036	0.221
Hyperactivity-inattention, median (IQR)	7 (5.75, 9)	7 (5, 8)	0.302	0.109
Peer problems, median (IQR)	5 (4, 7.25)	5 (4, 7)	0.687	0.042
Conduct problems, median (IQR)	2 (1, 3.25)	2 (1, 3)	0.759	0.032
Prosocial behavior, median (IQR)	3 (2, 4)	3.5 (1.25, 6)	0.474	0.075
Total scores, median (IQR)	17.5 (14.5, 23.25)	15 (13, 19)	0.106	0.170

Autism-related symptoms include ASD symptom severity, social deficits, and emotional and behavioral problems. Social deficits were evaluated by the Social Responsiveness Scale Second Edition (SRS-2). Emotional and behavioral problems were evaluated by the SDQ. Effect size = Z/√(N) for Mann–Whitney *U*-tests.

*ASD* autism spectrum disorder, *IQR* interquartile range.

Furthermore, a multivariate generalized linear model was employed to compare autism-related symptoms between the constipation and non-constipation groups of children with ASD, based on the results of the univariate analysis. The results showed that the scores for ASD symptom severity, social awareness, social communication, and SRS-2 total scores in the ASD constipation group were significantly higher than those in the non-constipation group (all *P-*values < 0.05, effect sizes > 0.1). Moreover, the scores for emotional symptoms and SDQ total scores in the constipation group were significantly higher than those in the non-constipation group of children with ASD (all *P-*values < 0.05, effect sizes > 0.15), as in Table 3.

**Table 3:** Covariate-adjusted comparisons of autism-related symptoms between the constipation and non-constipation groups of children with ASD (*n* = 90)

Variables	Adjusted β coefficient (95% CI)	*P-*value	Effect size
ASD symptom severity	2.600 (0.081 to 5.119)	0.043	1.404
Social deficits			
Social awareness	1.879 (0.407 to 3.350)	0.013	0.366
Social communication	5.185 (0.800 to 9.571)	0.021	0.912
Total scores	13.315 (1.388 to 25.242)	0.029	0.661
Emotional and behavioral problems			
Emotional symptoms	0.972 (0.122 to 1.823)	0.026	0.215
Total scores	2.652 (0.432 to 4.872)	0.020	0.555

Autism-related symptoms include ASD symptom severity, social awareness, social communication, social responsive scale-second edition (SRS-2) total scores, emotional symptoms, and SDQ total scores. Adjusted for child’s age, sex, intellectual functioning, advanced maternal age, feeding patterns before 2 years of age, daily sleep duration, daily moderate-to-vigorous physical activity (MVPA) duration, and parenting behavior. Effect size = Cohen’s f^2^ for linear models.

*ASD* autism spectrum disorder, *CI* confidence interval.

### Comparisons of gut microbiota in fecal samples between the constipation and non-constipation groups of children with ASD

**Fig. 1:**
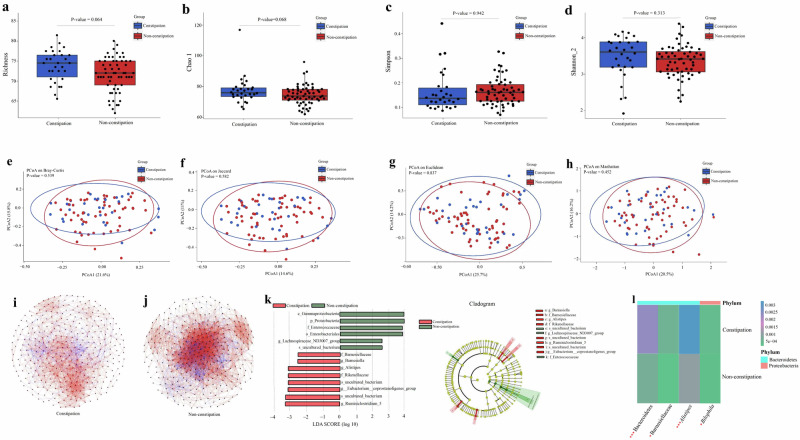
Altered gut microbiota between the constipation and non-constipation groups of children with ASD based on the 16S rRNA data (*n* = 90). **a**, **b** The Richness (**a**) and Chao1 (**b**) indices of gut bacterial taxa between the constipation and non-constipation groups of children with ASD at the genus level. **c**, **d** The Simpson (**c**) and Shannon_2 (**d**) indices of gut bacterial taxa between the constipation and non-constipation groups of children with ASD at the genus level. Statistically significant false discovery rate (FDR) correction, *P*-value < 0.05. **e**–**h** Principal coordinates analysis (PCoA) of the bacterial taxa based on Bray–Curtis distance (**e**), Jaccard distance (**f**), Euclidean distance (**g**), and Manhattan distance (**h**). Statistically significant *P*-value < 0.05. **i**, **j** Correlation network analysis of gut bacterial taxa in the ASD constipation group (**i**) and ASD non-constipation group (**j**). The network analysis was conducted based on the Spearman correlation algorithm. Each node presents one Operational Taxonomic Unit (OTU). The straight line represents a significant correlation (|*r*| > 0.4, FDR-corrected *P*-value < 0.05) between two nodes. Red: a positive correlation; Blue: a negative correlation. **k** Linear discriminant analysis (LDA) effect size (LEfSe) analysis between the constipation and non-constipation groups of children with ASD. LDA scores showed differentially abundant bacterial taxa between the ASD constipation group and the ASD non-constipation group (LDA > 2.5). Cladograms generated by LEfSe indicated differences in the bacterial taxa between the ASD constipation group and the ASD non-constipation group. Red bars represent taxa enrichment in the ASD constipation group, and green bars indicate taxa enrichment in the ASD non-constipation group, *P*-value < 0.05. **l** Significantly different in the relative abundance of gut bacterial taxa between the ASD constipation group and the ASD non-constipation group were tested using the Wilcoxon rank-sum at the phylum, family, and genus levels. ^*^FDR corrected *P*-value < 0.25, ^**^FDR corrected *P*-value < 0.1, ^***^FDR corrected *P*-value < 0.05. ASD autism spectrum disorder, 16S rRNA 16S ribosomal RNA.

For the alpha-diversity, the genus-level Richness, Chao1, Simpson, and Shannon_2 indices did not show statistically significant differences between the constipation and non-constipation groups of children with ASD [Wilcoxon rank-sum test, false discovery rate (FDR) adjusted *P-*value = 0.064, FDR-corrected *P-*value = 0.068, FDR-corrected *P-*value = 0.942, FDR-R-corrected *P-*value = 0.313)], as depicted in Fig. 1a–d.

In terms of beta diversity, the principal coordinate analysis (PCoA) binding permutational multivariate analysis of variance (PERMANOVA) test, calculated using Bray-Curtis distance, Jaccard distance, Euclidean distance, and Manhattan distance, showed no statistically significant difference between constipated and non-constipated children with ASD (*P-*value = 0.539, *P-*value = 0.582, *P-*value = 0.837, *P-*value = 0.452), as in Fig. 1e–h.

To further elucidate the differences in the interaction patterns of gut bacterial communities between the constipation and non-constipation groups of children with ASD, we conducted a comparative analysis of significantly correlated gut bacterial taxa. The results revealed that the number of significantly correlated gut bacterial taxa in the constipation group of ASD children (*r* < −0.4 > or *r* > 0.4, FDR-corrected *P*-values < 0.05) was strikingly lower compared to that in the non-constipation group of ASD children, as presented in Fig. 1i, j.

The linear discriminant analysis (LDA) effect size (LEfSe) analysis identified discriminatory bacterial signatures between groups, using the LDA threshold score of ≥2.5. Specifically, children in the ASD constipation group were characterized by significantly higher levels of Bacteroidetes and Firmicutes (all FDR-corrected *P*-values < 0.05), whereas the ASD non-constipation group exhibited elevated levels of Firmicutes and Proteobacteria (all FDR-corrected *P*-values < 0.05), as detailed in Fig. 1k.

Next, comparative analysis using Wilcoxon rank-sum tests revealed that the relative abundance of four gut bacterial taxa—including the phylum Bacteroidetes, the family Barnesiellaceae, and the genera *Alistipes* and *Bilophila*—was significantly elevated in constipated ASD children compared to their non-constipated peers (FDR corrected *P*-values < 0.25), as in Fig. 1l.

### The correlation between constipation and altered gut microbiota and autism-related symptoms in children with ASD

**Fig. 2:**
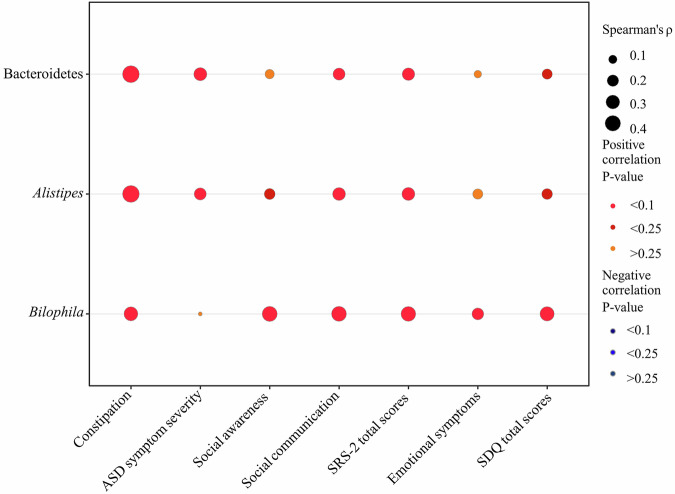
The correlation between constipation and altered gut microbiota and autism-related symptoms in children with ASD (*n* = 90). Constipation was evaluated by subscale scores of the 6-GSI. Autism-related symptoms include ASD symptom severity, social awareness, social communication, SRS-2 total scores, emotional symptoms, and SDQ total scores. The size of the bubble represents the correlation levels. The color of the bubble represents the *P*-value levels. Adjusted for child’s age, sex, intellectual functioning, advanced maternal age, feeding patterns before 2 years of age, daily sleep duration, daily MVPA duration, and parenting behavior. ASD, autism spectrum disorder.

This study analyzed the correlations between constipation and altered gut microbiota and autism-related symptoms in children with ASD. Constipation positively correlated with the abundances of the phylum Bacteroidetes and the genera *Alistipes* and *Bilophila* (*r* = 0.479, *P*-value < 0.001; *r* = 0.472, *P*-value < 0.001; *r* = 0.302, *P*-value = 0.006), as in Fig. 2 and Supplementary Table 1 for details. Second, there was a significant positive relationship between the relative abundances of gut bacterial taxa—namely Bacteroidetes, *Alistipes*, and *Bilophila*—and autism-related symptoms. The specific correlation results were presented in Fig. 2 and Supplementary Table 2. Bacteroidetes positively associated with ASD symptom severity (*r* = 0.262, *P-*value = 0.020) and SRS-2 totals (*r* = 0.234, *P-*value = 0.043); *Alistipes* positively associated with social communication (*r* = 0.253, *P-*value = 0.030) and SRS-2 totals (*r* = 0.257, *P-*value = 0.026); *Bilophila* demonstrated associations with social awareness (*r* = 0.362, *P-*value = 0.001), social communication (*r* = 0.356, *P-*value = 0.002), SRS-2 total scores (*r* = 0.347, *P-*value = 0.002), and SDQ total scores (*r* = 0.320, *P-*value = 0.005).

### Moderating effects of altered gut microbiota on the relationship between constipation and autism-related symptoms in children with ASD

**Fig. 3:**
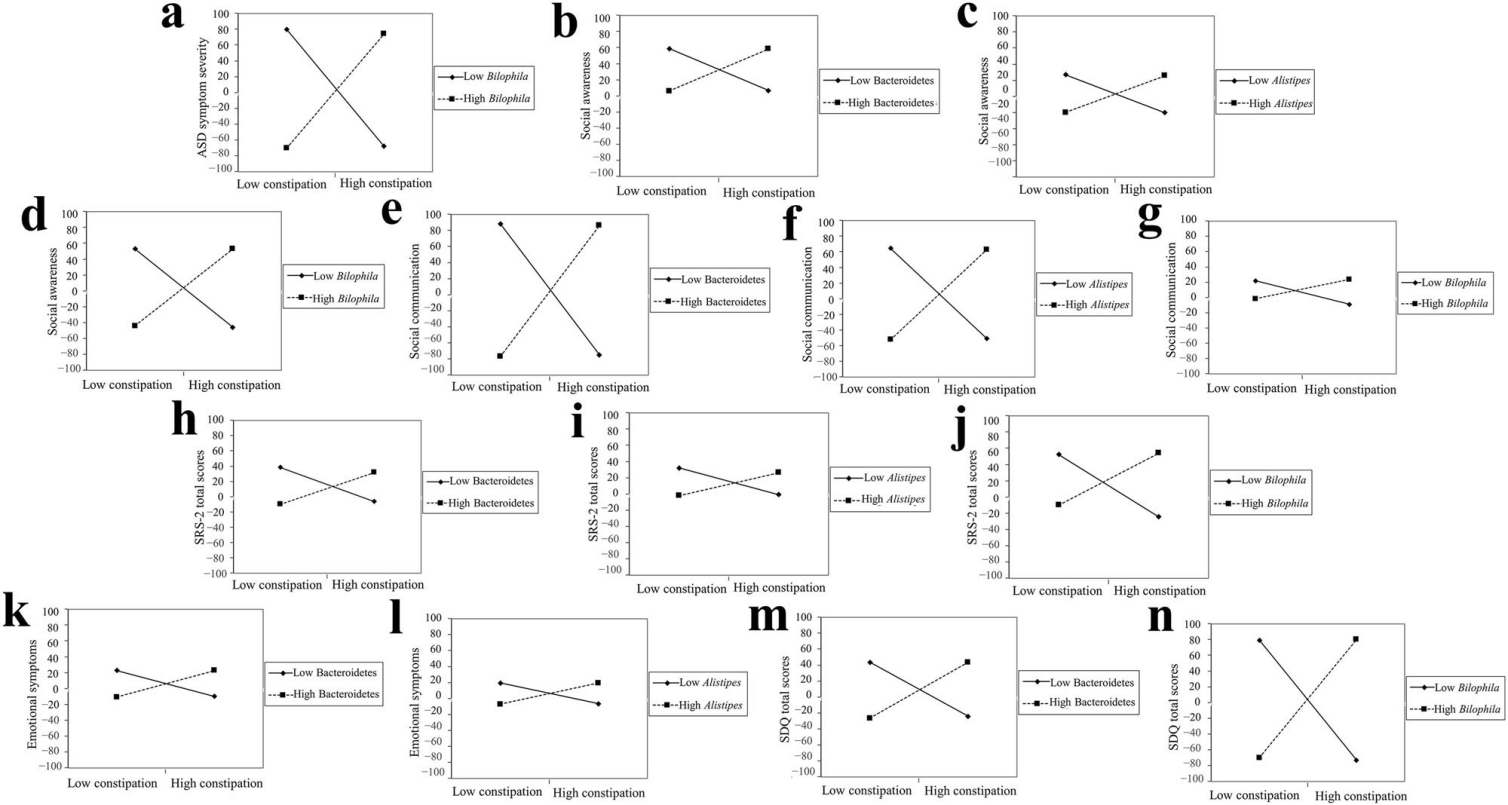
The altered gut microbiota exacerbated the relationship between constipation and autism-related symptoms in children with ASD (*n* = 90). Constipation was evaluated by subscale scores of the 6-GSI. Autism-related symptoms include ASD symptom severity, social awareness, social communication, SRS-2 total scores, emotional symptoms, and SDQ total scores. Adjusted for child’s age, sex, intellectual functioning, advanced maternal age, feeding patterns before 2 years of age, daily sleep duration, daily MVPA duration, and parenting behavior. ASD, autism spectrum disorder.

The results of moderating effects showed that *Bilophila* exacerbated the relationship between constipation and ASD symptom severity (*P*-value = 0.043). Bacteroidetes, *Alistipes*, and *Bilophila* exacerbated the relationship between constipation and social awareness (*P*-value = 0.030, *P*-value = 0.033, and *P*-value = 0.032). Bacteroidetes, *Alistipes*, and *Bilophila* exacerbated the relationship between constipation and social communication (*P*-value = 0.020, *P*-value = 0.037, and *P*-value = 0.029). Bacteroidetes, *Alistipes*, and *Bilophila* exacerbated the relationship between constipation and the SRS-2 total scores (*P*-value = 0.022, *P*-value = 0.038, and *P*-value = 0.028). Bacteroidetes and *Alistipes* exacerbated the relationship between constipation and emotional symptoms (*P*-value = 0.028 and *P*-value = 0.030). Bacteroidetes and *Bilophila* exacerbated the relationship between constipation and the SDQ total score (*P*-value = 0.087 and *P*-value = 0.035). See details in Table 4 and Fig. 3.

**Table 4:** The altered gut microbiota exacerbated the relationship between constipation and autism-related symptoms in children with ASD (*n* = 90)

Variables	Moderator	β	SE	95% CI	Value of t	*P*-value	Effect size
ASD symptom severity	*Bilophila*	7.294	3.538	0.254 to 14.334	2.061	0.043	0.927
Social awareness	Bacteroidetes	0.260	0.117	0.026 to 0.495	2.221	0.030	0.580
Social awareness	*Alistipes*	0.232	0.106	0.019 to 0.445	2.183	0.033	0.560
Social awareness	*Bilophila*	4.897	2.236	0.430 to 9.365	2.191	0.032	0.502
Social communication	Bacteroidetes	0.816	0.344	0.131 to 1.501	2.372	0.020	0.751
Social communication	*Alistipes*	0.576	0.271	0.036 to 1.115	2.125	0.037	0.724
Social communication	*Bilophila*	14.006	6.284	1.478 to 26.533	2.229	0.029	0.842
SRS-2 total scores	Bacteroidetes	2.163	0.926	0.318 to 4.008	2.336	0.022	0.572
SRS-2 total scores	*Alistipes*	1.538	0.727	0.090 to 2.986	2.114	0.038	0.536
SRS-2 total scores	*Bilophila*	35.143	16.660	1.947 to 68.338	2.109	0.038	0.603
Emotional symptoms	Bacteroidetes	0.165	0.074	0.019 to 0.312	2.247	0.028	0.305
Emotional symptoms	*Alistipes*	0.132	0.060	0.013 to 0.250	2.215	0.030	0.309
SDQ total scores	Bacteroidetes	0.344	0.198	–0.052 to 0.740	1.736	0.087	0.802
SDQ total scores	*Bilophila*	7.554	3.516	0.533 to 14.575	2.149	0.035	0.800

Constipation was evaluated by subscale scores of the 6-GSI. Autism-related symptoms include ASD symptom severity, social awareness, social communication, SRS-2 total scores, emotional symptoms, and SDQ total scores. Adjusted for child’s age, sex, intellectual functioning, advanced maternal age, feeding patterns before 2 years of age, daily sleep duration, daily MVPA duration, and parenting behavior. Effect size = Cohen’s f^2^ for moderating effect models.

*ASD* autism spectrum disorder, *SE* standard error, *CI* confidence interval.

### Comparisons of gut metabolites in fecal samples between the constipation and non-constipation groups of children with ASD

**Fig. 4:**
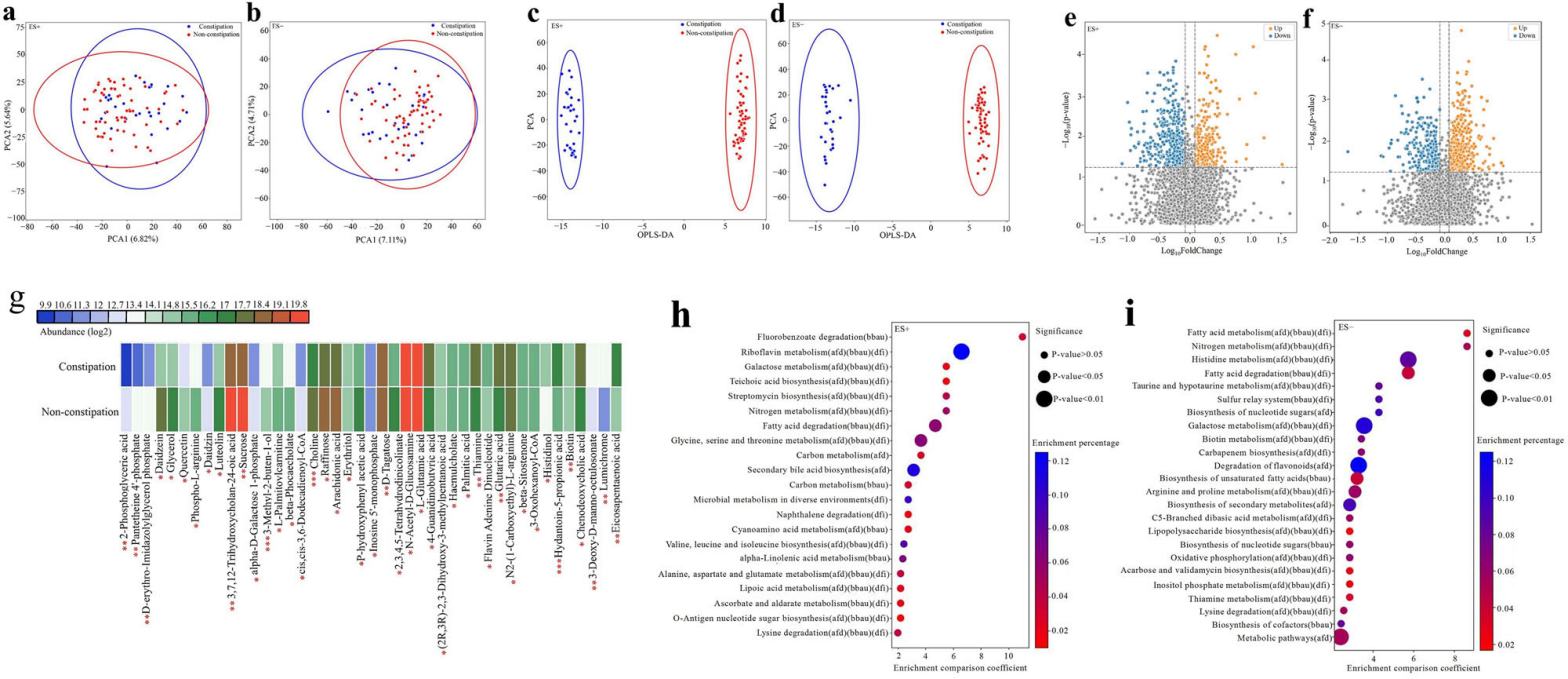
Altered gut metabolites between the constipation and non-constipation groups of children with ASD based on the UPLC-MS analysis (*n* = 90). **a**, **b** Principal component analysis (PCA) in the positive (**a**) and negative (**b**) ion modes. **c**, **d** Orthogonal partial least-squares discriminant analysis (OPLS-DA) in the positive (**c**) and negative (**d**) ion modes. **e**, **f** Volcano plot of the significantly differential gut metabolites in the positive (**e**) and negative (**f**) ion modes. The differential metabolites were screened out according to the fold change (FC) < 0.8 or >1.2 and *P*-value < 0.05. **g** Heatmap of the log2-transformed relative abundance of the differential gut metabolites [FC < 0.8 or > 1.2, variable importance in projection (VIP) > 1, and *P*-value < 0.05]. ^*^P-value < 0.05, ^**^P-value < 0.01, ^***^P-value < 0.001. **h**, **i** Bubble diagram of the KEGG pathways enrichment analyses in the positive (**h**) and negative (**i**) ion modes. The pathways were screened according to the significantly differential metabolites (FC < 0.8 or >1.2, VIP > 1, and *P*-value < 0.05). The bubble size indicates the *P*-value levels (computed using the hypergeometric distribution). The color of the bubble represents the enrichment percentage of the pathways. ASD autism spectrum disorder, UPLC-MS ultra-high-performance liquid chromatography-mass spectrometry, bbau bacteroidetes, afd *Alistipes*, dfi *Bilophila*.

Firstly, the principal component analysis (PCA) and orthogonal partial least squares-discriminant analysis (OPLS-DA) revealed the overall differences in gut metabolites between the constipation and non-constipation groups of children with ASD (Fig. 4a–d). Next, the significantly differential gut metabolites [fold change (FC) < 0.8 or > 1.2, *P*-value < 0.05] between the ASD constipation group and non-constipation group were visualized by the volcano plots, as in Fig. 4e, f. In addition, the top 43 differential gut metabolites were displayed via heatmap analysis between the two groups of ASD children [FC < 0.8 or > 1.2, variable importance in projection (VIP) > 1, *P*-value < 0.05], see Fig. 4g. Metabolite pathway enrichment analysis (MPEA) was performed on the significantly differential metabolites (screened by FC < 0.8 or > 1.2, VIP > 1, and *P*-value < 0.05 from the 451 detected metabolites) to identify key Kyoto Encyclopedia of Genes and Genomes (KEGG) pathways, which revealed a distinct metabolic profile between the constipation and non-constipation groups of children with ASD, as shown in Fig. 4h, i.

### The correlation between altered gut microbiota and gut metabolites in children with ASD

**Fig. 5:**
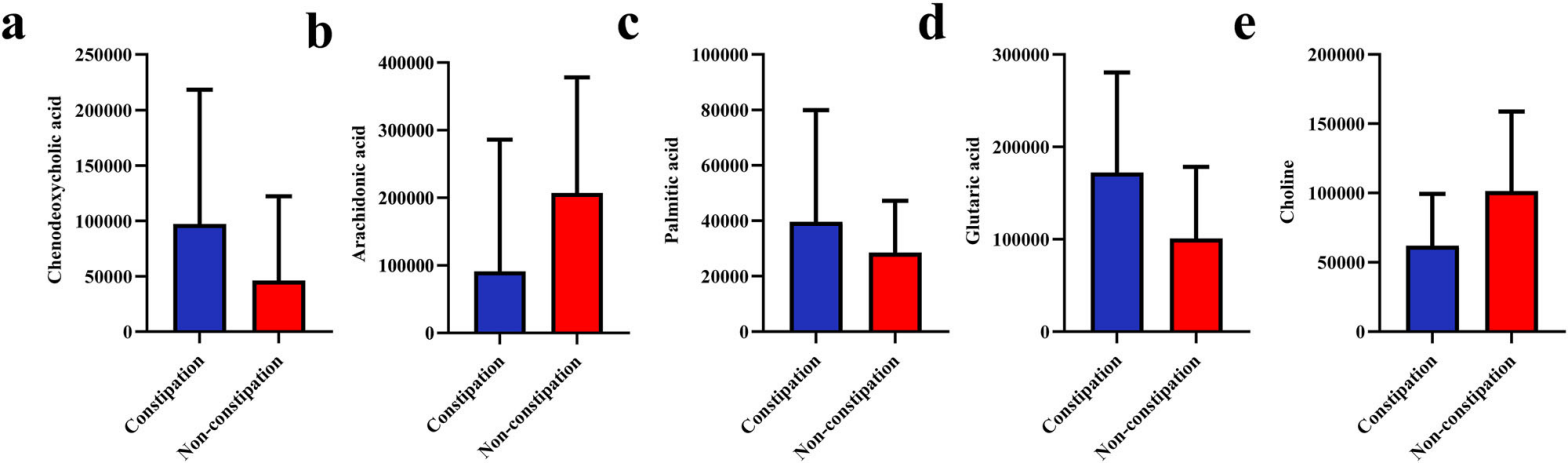
Histogram of the altered gut metabolites between the constipation and non-constipation groups of children with ASD (*n* = 90). The data were presented as median (IQR). These significantly altered gut metabolites between the constipation and non-constipation groups of children with ASD were defined by an FC < 0.8 or >1.2, VIP > 1, and a *P*-value < 0.05. ASD autism spectrum disorder, IQR interquartile range, FC fold change, VIP variable importance in projection.

**Fig. 6:**
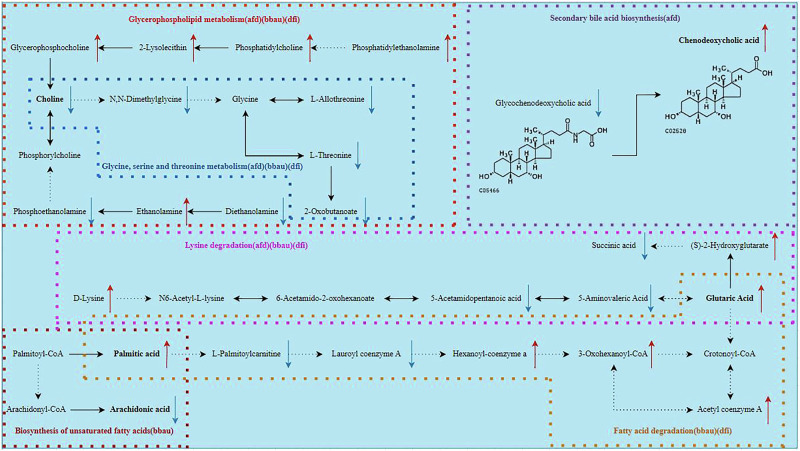
Five altered gut metabolites correlated with autism-related symptoms in the five KEGG pathways. The direct pathway according to the KEGG database. The indirect pathway according to the KEGG database. Decreased gut metabolites, FC < 0.8, VIP > 1, and *P*-value < 0.05. Increased gut metabolites, FC > 1.2, VIP > 1, and *P*-value < 0.05. Five altered gut metabolites derived from microbiota (Bacteroidetes, *Alistipes*, and *Bilophila*) were screened out, enriched in five metabolic pathways, and identified to correlate with autism-related symptoms in ASD children. ASD autism spectrum disorder, KEGG Kyoto Encyclopedia of Genes and Genomes, FC fold change, VIP variable importance in projection, bbau Bacteroidetes, afd *Alistipes*, dfi *Bilophila*.

Among the altered gut metabolites between the ASD constipation and non-constipation group, five altered gut metabolites (FC < 0.8 or >1.2, VIP > 1, *P*-value < 0.05) were identified to derive from the altered gut bacterial taxa (the phylum Bacteroidetes and the genera *Alistipes* and *Bilophila*) via KEGG pathways and MPEA. We observed three elevated metabolites (chenodeoxycholic acid, palmitic acid and glutaric acid) and and two reduced metabolites (arachidonic acid and choline) (Fig. 5). These metabolites mapped to five enriched pathways: secondary bile acid biosynthesis, unsaturated fatty acid biosynthesis, fatty acid degradation, lysine degradation, and glycerophospholipid metabolism (Fig. 6). Notably, chenodeoxycholic acid is a metabolite exclusively derived from a specific metabolic pathway of the bacterial genus *Alistipes*, while arachidonic acid is solely produced via a distinct metabolic pathway of the bacterial phylum Bacteroidetes.

**Fig. 7:**
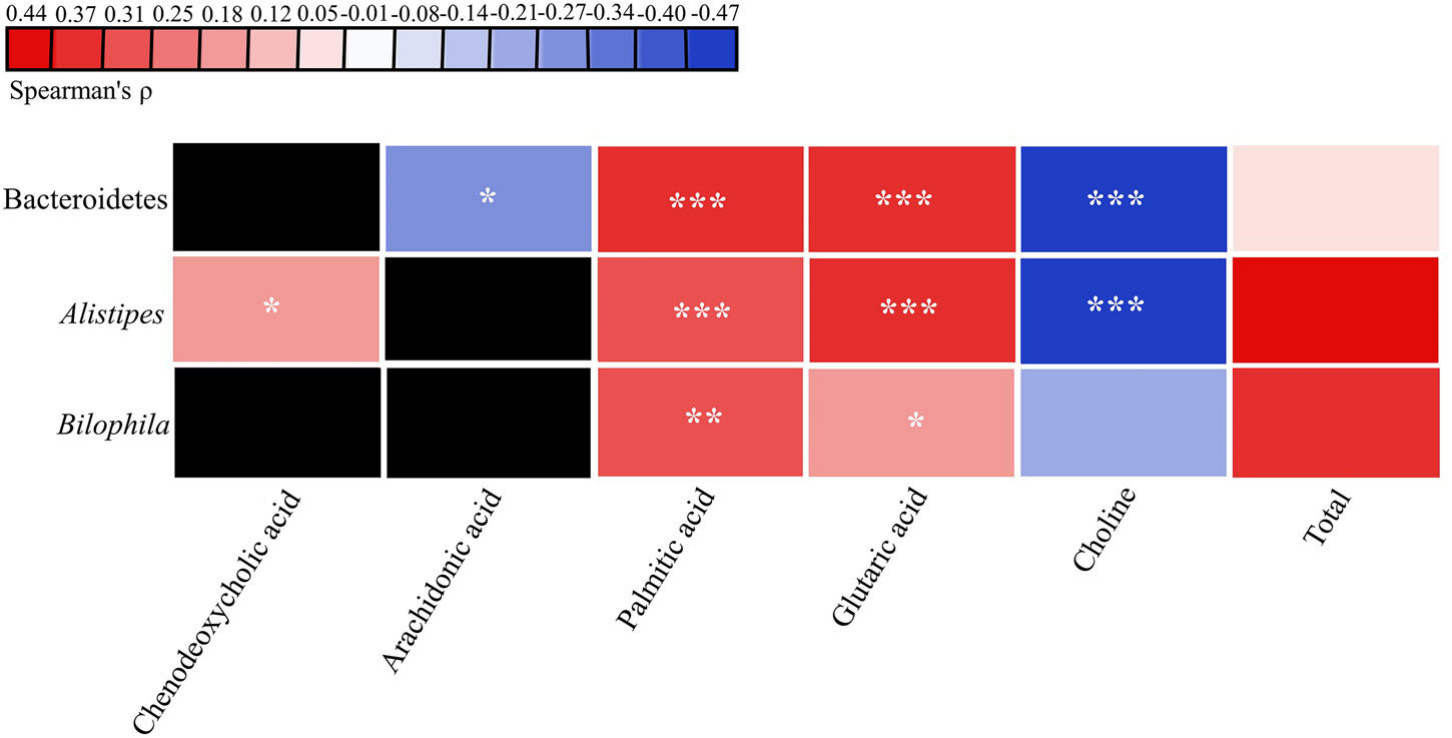
The correlation between altered gut microbiota and gut metabolites in children with ASD (*n* = 90). The “Total” on the right shows the total Spearman’s correlation coefficients between the altered gut bacterial taxa and gut metabolites. The altered gut metabolites were derived from the altered gut bacterial taxa according to the KEGG database. ^*^P-value < 0.05, ^**^P-value < 0.01, ^***^P-value < 0.001. ASD autism spectrum disorder.

This study found that three gut bacterial taxa with increased abundance in the ASD constipation group (compared to the non-constipation group)—Bacteroidetes, *Alistipes*, and *Bilophila*—were positively correlated with three elevated metabolites and negatively associated with two reduced metabolites, see Fig. 7 and Supplementary Table 3. Bacteroidetes positively correlated with palmitic acid and glutaric acid (*r* = 0.412, *P*-value < 0.001; *r* = 0.398, *P*-value < 0.001), but inversely correlated with arachidonic acid and choline (*r* = −0.26, *P*-value = 0.013; *r* = −0.458, *P*-value < 0.001). *Alistipes* positively correlated with chenodeoxycholic acid, palmitic acid, and glutaric acid (*r* = 0.225, *P*-value = 0.033; *r* = 0.364, *P*-value < 0.001; *r* = 0.382, *P*-value < 0.001) and a negative correlation with choline (*r* = −0.467, *P*-value < 0.001). *Bilophila* was positively correlated with palmitic acid and glutaric acid (*r* = 0.337, *P*-value = 0.001; *r* = 0.223, *P*-value = 0.034). It is worth noting that since chenodeoxycholic acid is exclusively produced by the gut bacterial genus *Alistipes* and arachidonic acid is solely derived from the gut bacterial phylum Bacteroidetes, the correlations between these metabolites and other gut bacterial taxa were not analyzed.

### The correlation between altered gut metabolites and autism-related symptoms in children with ASD

**Fig. 8:**
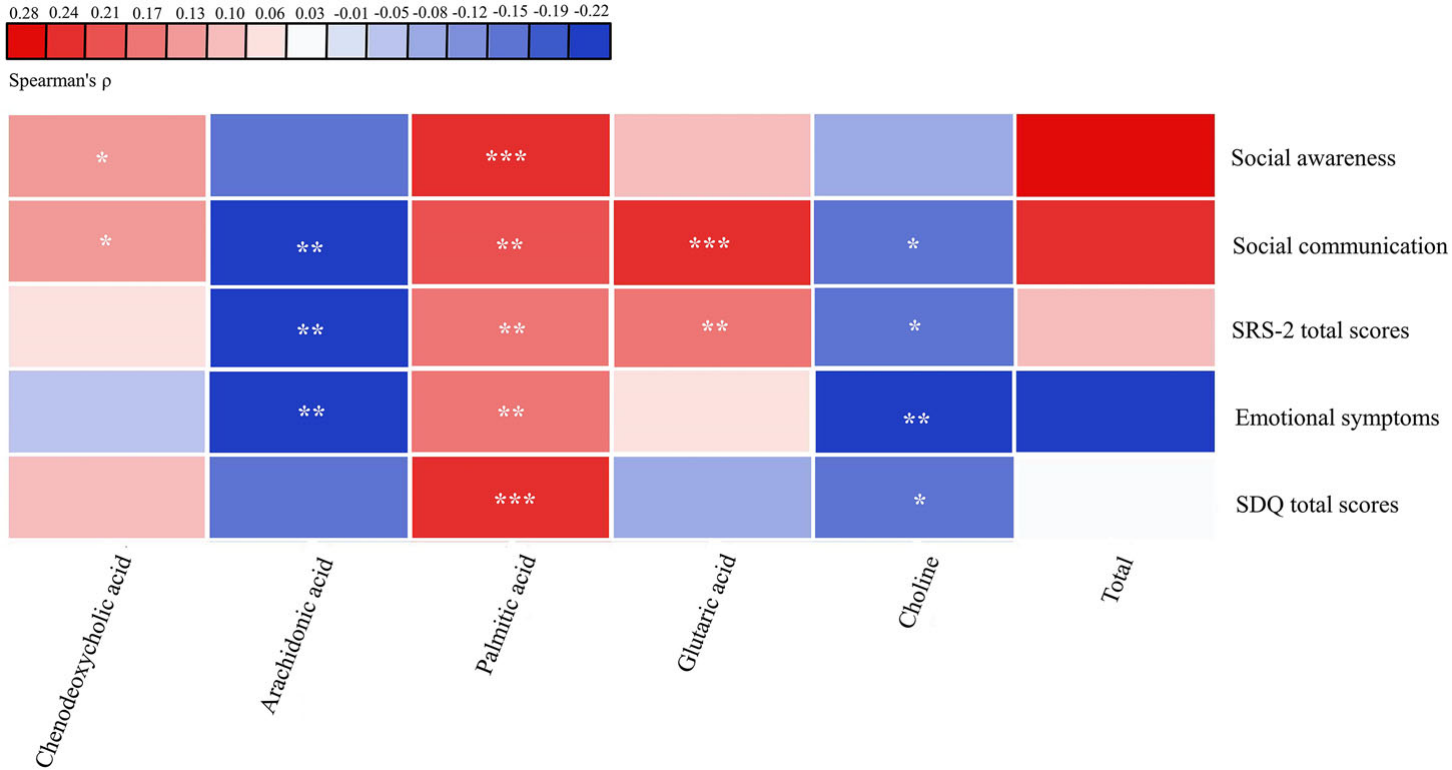
The correlation between altered gut metabolites and autism-related symptoms in children with ASD (*n* = 90). The “Total” on the right shows the total partial Spearman’s correlation coefficients between the altered gut metabolites and autism-related symptoms. Autism-related symptoms include ASD symptom severity, social awareness, social communication, SRS-2 total scores, emotional symptoms, and SDQ total scores. Adjusted for child’s age, sex, intellectual functioning, advanced maternal age, feeding patterns before 2 years of age, daily sleep duration, daily MVPA duration, and parenting behavior. ^*^P-value < 0.25, ^**^P-value < 0.1, ^***^P-value < 0.05. ASD autism spectrum disorder.

Among the altered gut metabolites between constipation and non-constipation groups of children with ASD, five altered gut metabolites derived from the altered gut bacterial taxa were significantly correlated with the autism-related symptoms, as detailed in Fig. 8 and Supplementary Table 4. Specifically, palmitic acid demonstrated a significant positive correlation with both social awareness and SDQ total scores (*r* = 0.260, *P*-value = 0.020; *r* = 0.244, *P*-value = 0.029), suggesting its potential role in exacerbating autism-related symptoms. Likewise, glutaric acid displayed a positive correlation with social communication (*r* = 0.249, *P*-value = 0.029). In contrast, choline exhibited an inverse relationship with emotional symptoms (*r* = −0.222, *P*-value = 0.039), indicating a potential protective effect against emotional difficulties.

### The correlation between constipation and altered gut metabolites in children with ASD

Constipation exhibited positive associations with three elevated gut metabolites, including chenodeoxycholic acid, palmitic acid, and glutaric acid (*r* = 0.252, *P*-value = 0.016; *r* = 0.236, *P*-value = 0.025; *r* = 0.310, *P*-value = 0.003, respectively). Conversely, negative correlations were observed with two decreased gut metabolites, specifically arachidonic acid and choline (*r* = −0.235, *P*-value = 0.025; *r* = −0.395, *P*-value < 0.001). Regarding the partial correlation between constipation and altered gut metabolites in children with ASD, constipation remained positively correlated with chenodeoxycholic acid, palmitic acid, and glutaric acid (*r* = 0.281, *P*-value = 0.011; *r* = 0.262, *P*-value = 0.018; *r* = 0.343, *P*-value = 0.002), while maintaining negative correlations with arachidonic acid and choline (*r* = −0.246, *P*-value = 0.027; *r* = −0.407, *P*-value < 0.001). Supplementary Fig. 1 and Tables 5 and 6.

## Discussion

This study revealed that constipated children with ASD demonstrated greater severity in autism-related symptoms—including ASD symptom severity, social deficits, and emotional-behavioral problems—than non-constipated ASD children. Four gut bacterial taxa (the phylum Bacteroidetes, the family Barnesiellaceae, and the genera *Alistipes* and *Bilophila*) exhibited significantly altered abundance in the constipated group relative to the non-constipated group of children with ASD. Among the four altered gut bacterial taxa, the phylum Bacteroidetes and the genera *Alistipes* and *Bilophila* exhibited elevated abundance in constipated ASD children, exacerbating the relationship between constipation and autism-related symptoms. Among gut metabolites altered between the constipation and non-constipation groups of children with ASD, five were derived from the altered gut bacterial taxa (the phylum Bacteroidetes and the genera *Alistipes* and *Bilophila*), as identified through KEGG pathways and MPEA. Additionally, these five gut metabolites—chenodeoxycholic acid, arachidonic acid, palmitic acid, glutaric acid, and choline—showed significant correlations with autism-related symptoms.

ASD children exhibit significantly higher GI symptom prevalence than TD peers, particularly constipation^1–4^. This study found that children in the ASD-constipation group exhibited more severe social deficits and emotional-behavioral problems than those in the non-constipation group. Multiple theoretical frameworks underlie the constipation-autism symptom nexus. One possible explanation is that GI issues may lead to physical discomfort and digestive problems, ultimately impacting social skills and emotional-behavioral problems in ASD children^17^. Another possibility is that dysbiosis in the gut microbiota may lead to abnormal release of metabolites that influence neuronal activity in the brain, thereby affecting social symptoms and emotional behavior in children with ASD^18^. Therefore, future research will explore the role of gut microbiota and metabolites in the relationship between constipation and autism-related symptoms in children with ASD.

This study showed that the ASD constipated group had significantly increased abundances of the phylum Bacteroidetes, the family Barnesiellaceae, and the genera *Alistipes* and *Bilophila* compared to the non-constipation group. Previous research has noted that Bacteroidetes abundance correlates with constipation severity^19^. High-fat diet may lead to an increase in Bacteroidetes in the intestinal tract^20^, thereby increasing the digestion time, reducing the speed of gastric emptying, and affecting the balance of gut bacterial taxa, thus aggravating the occurrence of constipation. Regarding Barnesiellaceae, a study analyzing the gut microbiome in a large population found the presence of Barnesiellaceae, though its specific role in GI function was not clarified^21^. As for *Alistipes*, a member of the Rikenellaceae family, studies have shown significant differences in gut bacterial taxa composition between children with ASD and TD children, specifically higher Rikenellaceae abundance in the ASD group^22^. Although no direct comparison between the ASD constipated and non-constipated groups was made regarding Rikenellaceae, these findings provide a foundation for further exploration of the relationship between constipation and Rikenellaceae in ASD. Other research has found a significant increase in *Alistipes* abundance in constipated individuals, with a positive correlation between *Alistipes* presence and constipation severity. This suggests that *Alistipes* may be associated with the occurrence and exacerbation of constipation^23^. *Bilophila*, a member of the Desulfovibrionaceae family, was shown in a double-blind, randomized, crossover intervention study to have a reduced relative abundance in constipated patients after consuming the prebiotic inulin. This reduction in *Bilophila* abundance was associated with softer stools and favorable changes in constipation-specific quality of life indicators^24^, highlighting the link between *Bilophila* abundance and constipation. Inflammatory bowel disease models in pigs have shown a reduction in bile acid metabolites and short-chain fatty acids, with increased presence of harmful microorganisms like *Bilophila* and *Alistipes*^25^, further supporting their pathogenic roles in gut inflammation.

This study found that the phylum Bacteroidetes was positively correlated with ASD symptom severity. The research revealed a significant alteration in the Firmicutes/Bacteroidetes ratio in autistic subjects due to an alteration of the Bacteroidetes relative abundance, suggesting that Bacteroidetes may play an important role in ASD pathogenesis^26^. Furthermore, our study found that the phylum Bacteroidetes and the genera *Alistipes* and *Bilophila* were positively correlated with social deficits, while the genus *Bilophila* was also associated with emotional-behavioral problems in children with ASD. The genus *Alistipe* has been shown to have a significant effect on diseases, including depression, anxiety, chronic fatigue syndrome, cirrhosis, and aging^27^. Another study has also reported an increase in the abundance of Bacteroidetes genera and some *Alistipes* in children with ASD^28^, and *Alistipes* is a known producer of propionate^27^. Dysbiosis in oral and gut microbiota, marked by altered levels of acetate, propionate, and butyrate, interferes with gut-brain and oral-brain connections, contributing to behavioral and neurological symptoms in ASD^29^. *Bilophila* has also been associated with Parkinson’s and Alzheimer’s diseases^30,31^, suggesting its potential involvement in neurodegenerative disease pathogenesis. Future research is needed to validate the relationship between *Bilophila* and ASD and to explore its role in ASD pathogenesis.

This study identified significant alterations in the abundances of gut microbiota-derived metabolites (chenodeoxycholic acid, arachidonic acid, palmitic acid, glutaric acid, and choline) in the ASD constipated group. Chenodeoxycholic acid can activate mast cells to release histamine and tryptase, which affect submucosal neuron excitability and regulate GI motility and constipation^32^. Arachidonic acid may regulate GI function by affecting gastric acid secretion and mucosal blood flow^33^. Prostaglandin E2 (PGE2), derived from arachidonic acid, protects the GI tract by inhibiting GI contractions, and preventing bacterial invasion^34^. In addition to its effects on individual cells, PGE2 also coordinates peristalsis by acting directly on the mesentery and myenteric plexus, leading to smooth muscle contraction and increased intraluminal pressure^35^. Reduced arachidonic acid levels in the ASD constipated group may lead to constipation and negatively affect GI health. Based on our findings and previous literature, increased palmitic acid abundance in the ASD constipated group may affect the mechanosensory apparatus of intestinal cells and thus contribute to constipation^36^. Current research on glutaric acid has predominantly focused on its pathological mechanisms in glutaric acidemia^37^, while its role in ASD and related GI issues has received limited attention. This gap warrants further exploration, building upon the findings of this study. Choline is essential for neuronal development, and its deficiency or overabundance has been linked to intestinal inflammation^38^. Studies have shown that the levels of choline or its derivatives were significantly associated with constipation and depression, suggesting that the interaction between gut bacterial taxa and choline metabolism may affect intestinal motility or inflammatory status^39^.

This study has several limitations. First, dividing ASD children into constipation and non-constipation groups led to a small sample size, reducing statistical power and potentially underestimating the number of altered gut bacterial taxa and gut metabolites. Future studies should use larger sample sizes to further investigate gut microbiota related to constipation in children with ASD. Second, this study used a cross-sectional design, preventing the examination of causal effects between constipation, gut microbiota, metabolites, and autism-related symptoms. Future research should employ longitudinal cohort designs or genetic studies to explore these relationships in depth. Lastly, this study only focused on the role of fecal sample metabolites, and future research should integrate blood metabolomics and proteomics to elucidate the mechanisms through which constipation exacerbates autism-related symptoms in children with ASD.

## Methods

### Study design and participants

**Fig. 9:**
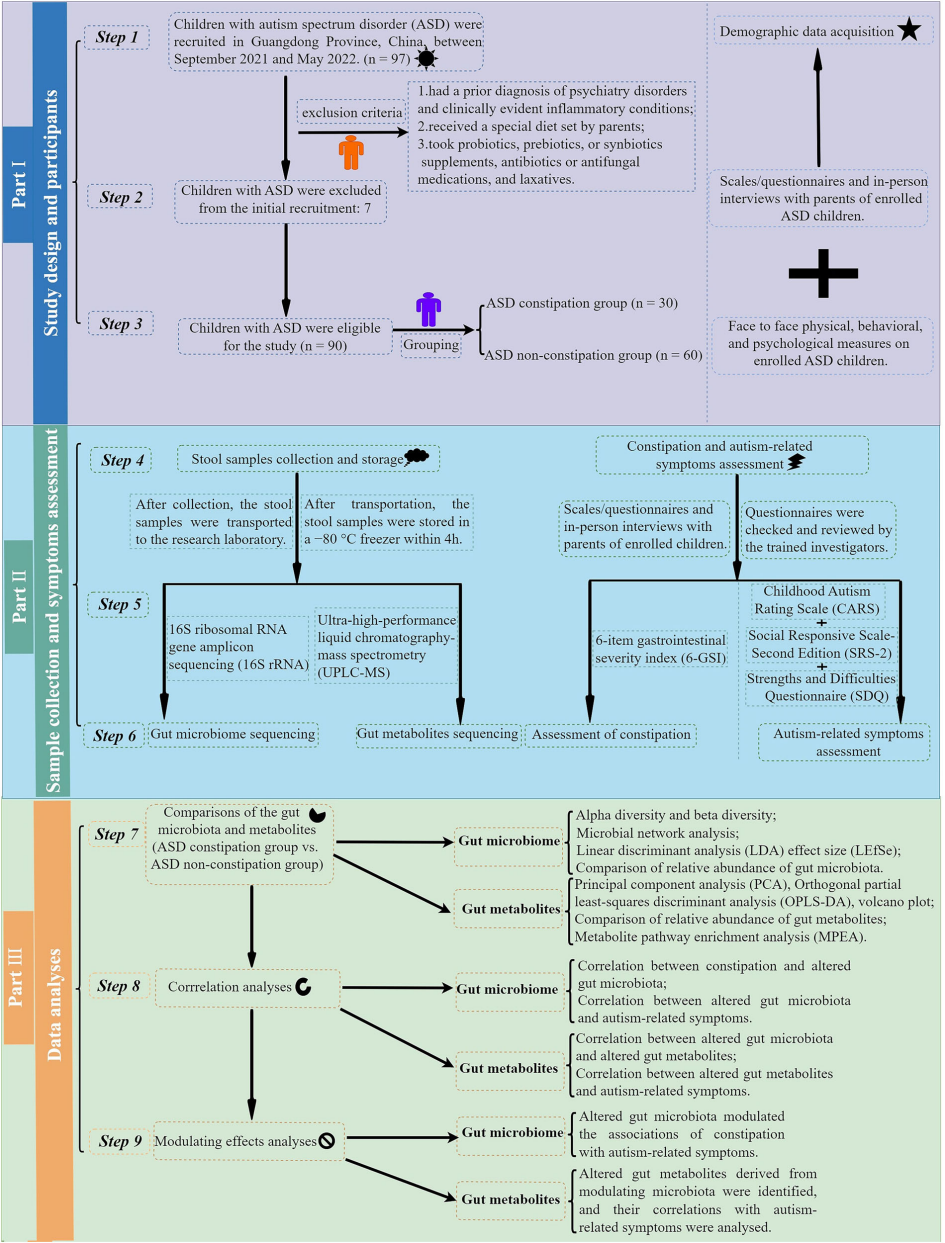
Flow chart of the study.

The research was conducted as a cross-sectional study. Children with ASD from 2 to 10 years old were consecutively recruited from Guangzhou, Guangdong Province, China, between September 2021 and May 2022, as shown in Fig. 9. The diagnoses of ASD were established via the Autism Diagnosis Observation Schedule-2 (ADOS-2)^40^ following the Diagnostic and Statistical Manual of Mental Disorders, 5th Edition (DSM-5)^41^ criteria. The inclusion criteria were applied to ASD children: (a) normal vision (or corrected vision) and normal hearing; (b) only the first child will be included if there are two or more eligible children with ASD in one family. The exclusion criteria were described in the previous study and displayed in Fig. 9^42^. The study finally enrolled a total of 90 children with ASD based on the strict inclusion and exclusion criteria. Based on the six-item Gastrointestinal Severity Index (6-GSI) questionnaire, ASD children were stratified into constipation and non-constipation groups according to the number of bowel movements per week.

To collect demographic and behavioral symptom data, the validated scales/questionnaires were used, followed by in-person interviews with parents of enrolling children with ASD. Also, the face-to-face physical, behavioral, and psychological measurements were performed in enrolling children with ASD. Next, the participants provided their fecal samples for microbiome and metabolomic analysis. The study was conducted in accordance with the principles of the Declaration of Helsinki and was approved by the Ethical Committee of the School of Public Health, Sun Yat-Sen University (No. 067 [2022]). The written informed consent was duly obtained from the parents.

### GI symptoms assessments

A shortened version of the 6-GSI questionnaire was designed to index the severity of GI symptoms in children with ASD^43^. The sensitivity and specificity for identifying GI problems in children with ASD were 80% and 79%, respectively^44^. The 6-GSI assesses six symptoms: constipation, diarrhea, abnormal stool consistency, abnormal stool odor, abdominal distention, and abdominal pain. Each symptom is rated on a three-point Likert-type scale ranging from 0 to 2. The constipation group of children with ASD (*n* = 30 ASD) had 0–2 bowel movements per week. The non-constipation group (*n* = 60 ASD) had 5 or more bowel movements per week.

### ASD symptom severity

ASD symptom severity is clinically evaluated using the Childhood Autism Rating Scale (CARS)^45^. The CARS has demonstrated strong internal consistency, reliable interrater and test-retest results, high criterion validity, and excellent discriminant validity^46^. The CARS is a clinician-rated questionnaire with a 4-point Likert scale based on observation of the individuals, as well as collateral information, such as parent or caregiver reports. It is comprised of 15 items covering a range of functions, including social, emotional, adaptive, communicative, and cognitive. Final rating scores from CARS are divided into 2 severity groups: mild-to-moderate symptoms of ASD (CARS score between 30 and 36.5) and severe symptoms of ASD (CARS score between 37 and 60)^45^.

### Social deficits

The SRS-2 is a parent-, teacher-, or caregiver-report screening instrument for the presence and severity of social deficits in children diagnosed with ASD^47^. The SRS-2 showed 96.9% sensitivity and 94.2% specificity for identifying the severity of impairments in social domains seen in ASD^48^. The 65-item SRS-2 uses a 4-point Likert scale (0 = never to 3 = always), providing a total score and five subscale scores: social awareness, cognition, communication, motivation, and autistic mannerisms. Higher scores denote more severe social deficits.

### Emotional and behavioral problems

The SDQ quantifies emotional and behavioral problems in children and teenagers^49^, demonstrating good validity and reliability^50^. It's 25 items from five subscales: emotional symptoms, hyperactivity, peer relationship problems, conduct problems, and prosocial behavior. Each item is rated on a 3-point Likert scale (0 for “Not True”, 1for “Somewhat True”, 2 for “Certainly True”), with domain scores ranging from 0 to 10. The score for total difficulties (ranging from 0 to 40) is the sum of all domain scores except for the prosocial behavior domain. A higher total score for emotional and behavioral problems reflects greater difficulty, while a higher score in the prosocial behavior domain indicates lesser difficulty.

### Fecal sample collection

Fecal samples were collected according to the instructions at home or the assessment center with a collection kit prepared in advance. After the collection period, the fecal samples were shipped using dry ice by The City Delivery to the research laboratory and stored in a −80 °C freezer within 4 h for subsequent 16S rRNA gene sequencing and non-targeted metabolomics.

### 16S rRNA gene amplicon sequencing

16S rRNA gene amplicon sequencing followed established methods^42^. Fecal DNA was extracted with QIAamp DNA Stool Mini Kit (QIAGEN), with quality (integrity/purity/concentration) assessed via Nanodrop spectrophotometer (Nanodrop Technologies). PCR templates were diluted to 2 ng/μL. The primer pair of 338F (5′-ACTCCTACGGGAGGCAGCA-3′) and 806 R (5′-GGACTACHVGGGTWTCTAAT-3′) was used to amplify the V3–V4 region of the bacterial 16S rRNA gene. PCR reactions were performed in triplicate using BioRad S1000 (Bio-Rad Laboratory, Hercules, CA, USA). High-throughput sequencing was performed on the Illumina HiSeq 2500 platform (MAGIGENE Co., Ltd., Guangzhou, China). The sequencing reads were merged using USEARCH (version 10.0.240)^51^ and quality-filtered by fastp (version 0.14.1)^52^. Sequences were clustered at a 97% similarity cutoff value by UPARSE (V11)^53^ to generate operational taxonomic units (OTUs), and were subjected to singleton removal and rarefaction before subsequent analysis. At last, a taxonomic assignment was classified using the RDP Bayes-Classifier^54^ with a confidence threshold of 80%.

Alpha diversity metrics (Richness, Chao 1, Simpson, and Shannon_2) were evaluated at the genus level with the Wilcoxon rank-sum tests. Beta diversity patterns were examined through PCoA using four distance metrics (Bray–Curtis, Jaccard, Euclidean, Manhattan), complemented by PERMANOVA testing through R’s vegan package^55^. A microbial interaction network was constructed from the 373 most abundant OTUs using Spearman correlation (|r| > 0.4, FDR corrected *P*-value < 0.05), visualized through Gephi^56^ with node sizes reflecting OTU relative abundance. Bacterial taxa-specific differences were identified between the ASD constipation group and the ASD non-constipation group through the LEfSe analysis (LDA > 2.5)^57^ and validated by Wilcoxon rank-sum tests (at the phylum, family, and genus levels) with Benjamini–Hochberg (BH) correction.

### Untargeted metabolomic analysis using UPLC-MS

The Ultra Performance Liquid Chromatography Mass Spectrometry (UPLC-MS) system (Agilent Technologies, Santa Clara, CA, USA) was described in a previous publication^42^. Briefly, chromatographic separation utilized a Waters Acquity UPLC HSS T3 C18 column (2.1 mm × 100 mm, 1.8 μm) at 40 °C and a flow rate of 0.4 mL/min. Mobile phases were 0.1% formic acid in H_2_O (A) and 0.1% formic acid in acetonitrile (B). Mass spectrometry analyzed samples in alternating positive/negative ionization modes via time-of-flight mass spectrometry (TOF MS), scanning 100–1500 *m*/*z*. The electrospray ionization (ESI) parameters included Ion Source Gas1 (Gas1) at 50, Ion Source Gas2 (Gas2) at 60, curtain gas (CUR) at 35, a source temperature of 550 °C, and IonSpray Voltage Floating (ISVF) at ±5500/−4500 V. Raw LC-MS data were converted to mzML using ProteoWizard and processed in XCMS for peak extraction, alignment, and retention time correction. The support vector regression (SVR) refined peak areas, and peaks detected in <50% of samples per group were removed. Metabolite identification relied on an integrated database approach, combining in-house, public, artificial intelligence-driven, and metDNA resources.

PCA and OPLS-DA were conducted in both ionization modes to distinguish metabolic profile variations between the ASD constipation group and the ASD non-constipation group. Statistically significant metabolites (FC < 0.8 or >1.2, *P*-value < 0.05) were visualized via volcano plots generated with R/ggplot2 (v4.1.0). A heatmap highlighted log2-transformed abundance patterns of discriminatory metabolites meeting criteria (FC < 0.8 or >1.2, VIP > 1, *P*-value < 0.05). For pathway-level interpretation, MPEA was performed using R/clusterProfiler (v3.8.1) to identify the key KEGG pathways associated with significantly differential metabolites (FC < 0.8 or >1.2, VIP > 1, and *P*-value < 0.05). Bacterial taxa-associated metabolites were annotated against the KEGG compound database and mapped to biological pathways, with results displayed as bubble plots where bubble size reflected significance (*P*-value, hypergeometric test) and color gradient indicated pathway enrichment ratios.

### Statistical analyses

Non-normal continuous variables were presented as medians and interquartile ranges (IQRs), analyzed by Wilcoxon rank-sum tests; categorical variables employed counts (%) with chi-square tests. The multivariate generalized linear models were employed to investigate the relationship between constipation and autism-related symptoms in children with ASD. The associations between constipation and altered gut bacterial taxa and autism-related symptoms were quantified with Spearman’s correlation coefficients and related Spearman’s *P*-value. Finally, this study utilized the moderating models in the Process plugin to explore the moderating effects of gut bacterial taxa and their metabolites on relationships between constipation and autism-related symptoms in children with ASD (bootstrap 5000 iterations, α = 0.05).

In order to reduce bias and improve the reliability and stability of the results, this study controlled for multiple covariates, including the child’s age, sex, intellectual functioning, advanced maternal age, feeding patterns before 2 years of age, daily sleep duration, daily moderate-to-vigorous physical activity (MVPA) duration, and parenting behavior. Detailed information on the covariates was presented in previously published methods^42^. The collinearity diagnosis was applied to examine potential multicollinearity between variables in the models, including generalized linear models, Spearman’s correlation analysis, and moderating models. To estimate the effect size, the formula *r* = Z/√(N) was used for the Wilcoxon rank sum test, the effect size φ was used for 2 × 2 contingency tables, and the effect size Cramer’s V was used for multivariate contingency tables. The above effect sizes were characterized as small, medium, and large by boundary values > 0.1, >0.3, and >0.5, respectively^58^. Cohen’s *f*^2^ ≥ 0.02, *f*^2^ ≥ 0.15, and *f*^2^ ≥ 0.35 were used to characterize the effect sizes as small, medium, and large, respectively^59^.

All analyses were performed in R PROGRAMMING LANGUAGE (Version 4.1.0; R Foundation for Statistical Computing, Vienna, Austria) and SPSS Statistics 26.0 (IBM SPSS Statistics for Windows, Version 26.0. Armonk, NY, USA: IBM Corp). The Plots were drawn using R 4.1.0 and GraphPad Prism version 8.4.2 (GraphPad Software, San Diego, CA, USA). The two-tailed *P* value < 0.05 was considered statistically significant.

## Supplementary information

Supplement information

## Data Availability

All original data in this work have been deposited in the Science Data Bank (10.57760/sciencedb.25224). The data can also be obtained from the corresponding author upon reasonable request or from the included supplementary information files.
