# Supplementary material for: Multiomics analysis reveals the exacerbating effect of constipation on autism-related symptoms in children with autism spectrum disorder
Source: NPJ Biofilms Microbiomes. 2026 Jan 8;12:28. doi: 10.1038/s41522-025-00894-5 (PMC12852942; doi:10.1038/s41522-025-00894-5)
Supplement: Supplementary file 1 — Supplement information [file 41522_2025_894_MOESM1_ESM.pdf]

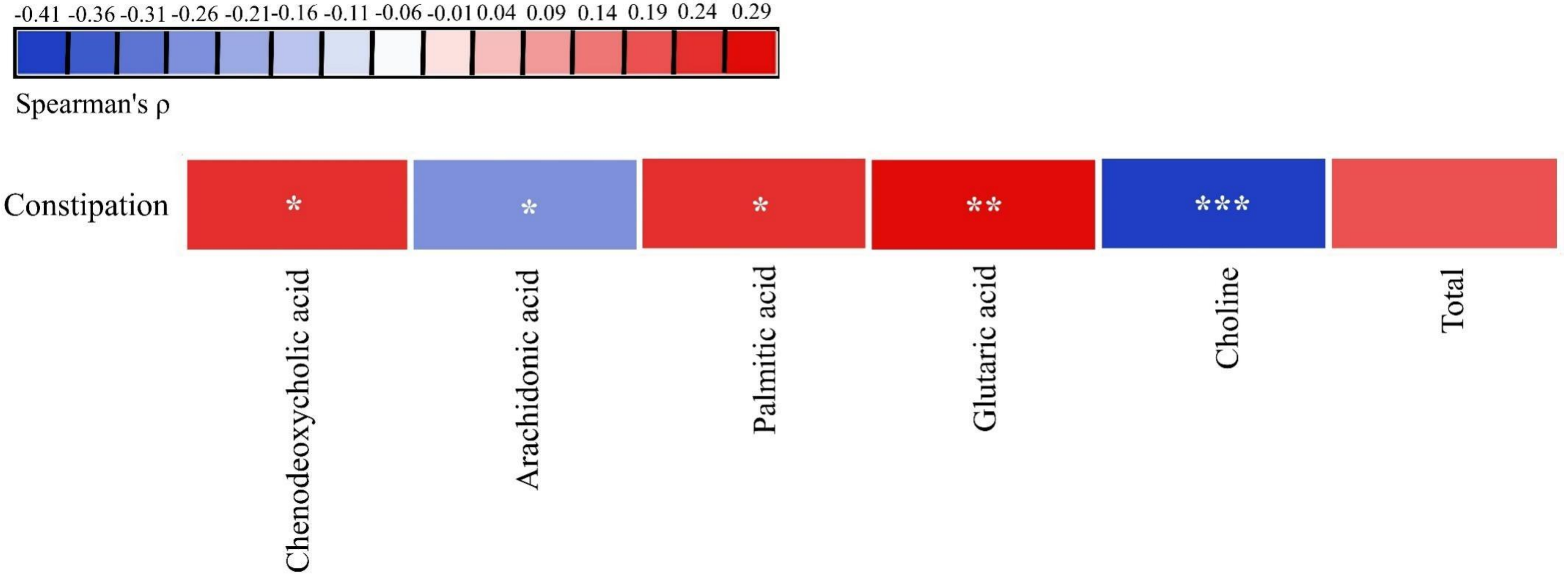

Supplementary Figure 1 The correlation between constipation and altered gut metabolites in children with ASD (n = 90).
Constipation was evaluated by subscale scores of the 6-item gastrointestinal severity index (6-GSI). The "Total" on the right shows the total partial Spearman's correlation coefficients between constipation and the five altered gut metabolites. Adjusted for child's age, sex, intellectual functioning, advanced maternal age, feeding patterns before 2 years of age, daily sleep duration, daily moderate-to-vigorous physical activity (MVPA) duration, and parenting behavior. * P-value < 0.05, ** P-value < 0.01, *** P-value < 0.001. ASD, autism spectrum disorder.

Supplementary Table 1 The correlation between constipation and altered gut microbiota in children with ASD (n = 90).

Variables	Altered gut bacterial taxa	Partial correlation coefficients	P-value	Negative_Positive
Constipation	Bacteroidetes	0.479	<0.001	Positive
Constipation	*Alistipes*	0.472	<0.001	Positive
Constipation	*Bilophila*	0.302	0.006	Positive

Constipation was evaluated by subscale scores of the 6-item gastrointestinal severity index (6-GSI). Adjusted for child's age, sex, intellectual functioning, advanced maternal age, feeding patterns before 2 years of age, daily sleep duration, daily moderate-to-vigorous physical activity (MVPA) duration, and parenting behavior. ASD, autism spectrum disorder.

Supplementary Table 2 The correlation between altered gut microbiota and autism-related symptoms in children with ASD (n = 90).

**Altered gut bacterial taxa**	**Autism-related symptoms**	**Partial correlation coefficients**	**P-value**	**Negative_Positive**
Bacteroidetes	ASD symptom severity	0.262	0.020	Positive
Alistipes	ASD symptom severity	0.215	0.054	Positive
Bilophila	ASD symptom severity	0.040	0.726	Positive
Bacteroidetes	Social awareness	0.118	0.305	Positive
Alistipes	Social awareness	0.169	0.142	Positive
Bilophila	Social awareness	0.362	0.001	Positive
Bacteroidetes	Social communication	0.218	0.064	Positive
Alistipes	Social communication	0.253	0.030	Positive
Bilophila	Social communication	0.356	0.002	Positive
Bacteroidetes	SRS-2 total scores	0.234	0.043	Positive
Alistipes	SRS-2 total scores	0.257	0.026	Positive
Bilophila	SRS-2 total scores	0.347	0.002	Positive
Bacteroidetes	Emotional symptoms	0.075	0.524	Positive
Alistipes	Emotional symptoms	0.145	0.262	Positive
Bilophila	Emotional symptoms	0.202	0.085	Positive
Bacteroidetes	SDQ total scores	0.149	0.210	Positive
Alistipes	SDQ total scores	0.166	0.153	Positive
Bilophila	SDQ total scores	0.320	0.005	Positive

Autism-related symptoms include ASD symptom severity, social awareness, social communication, social responsive scale-second edition (SRS-2) total scores, emotional symptoms, and strengths and difficulties questionnaire (SDQ) total scores. Adjusted for child's age, sex, intellectual functioning, advanced maternal age, feeding patterns before 2 years of age, daily sleep duration, daily moderate-to-vigorous physical activity (MVPA) duration, and parenting behavior. ASD, autism spectrum disorder.

Supplementary Table 3. The correlation between altered gut microbiota and gut metabolites in children with ASD (n = 90).

Altered gut bacterial taxa	Altered gut metabolites	Correlation coefficients	P-value	Negative_Positive
Bacteroidetes	Arachidonic acid	-0.26	0.013	Negative
	Palmitic acid	0.412	<0.001	Positive
	Glutaric acid	0.398	<0.001	Positive
	Choline	-0.458	<0.001	Negative
	Total	0.092		Positive
Alistipes	Chenodeoxycholic acid	0.225	0.033	Positive
	Palmitic acid	0.364	<0.001	Positive
	Glutaric acid	0.382	<0.001	Positive
	Choline	-0.467	<0.001	Negative
	Total	0.504		Positive
Bilophila	Palmitic acid	0.337	0.001	Positive
	Glutaric acid	0.223	0.034	Positive
	Choline	-0.16	0.132	Negative
	Total	0.4		Positive

The “Total” on the right shows the total Spearman's correlation coefficients between the altered gut bacterial taxa and gut metabolites. The gut metabolites were derived from the altered gut bacterial taxa according to the Kyoto Encyclopedia of Genes and Genomes (KEGG) database. ASD, autism spectrum disorder.

Supplementary Table 4. The correlation between altered gut metabolites and autism-related symptoms in children with ASD (n = 90).

Altered gut metabolites	Autism-related symptoms	Partial correlation coefficients	P-value	Negative_Positive
Chenodeoxycholic acid	Social awareness	0.136	0.231	Positive
	Social communication	0.141	0.217	Positive
	SRS-2 total scores	0.082	0.483	Positive
	Emotional symptoms	-0.040	0.738	Negative
	SDQ total scores	0.125	0.268	Positive
Arachidonic acid	Social awareness	-0.121	0.312	Negative
	Social communication	-0.213	0.071	Negative
	SRS-2 total scores	-0.225	0.051	Negative
	Emotional symptoms	-0.202	0.089	Negative
	SDQ total scores	-0.133	0.260	Negative
Palmitic acid	Social awareness	0.260	0.020	Positive
	Social communication	0.219	0.052	Positive
	SRS-2 total scores	0.210	0.063	Positive
	Emotional symptoms	0.189	0.088	Positive
	SDQ total scores	0.244	0.029	Positive
Glutaric Acid	Social awareness	0.102	0.381	Positive
	Social communication	0.249	0.029	Positive
	SRS-2 total scores	0.191	0.096	Positive
	Emotional symptoms	0.077	0.513	Positive
	SDQ total scores	-0.052	0.663	Negative
Choline	Social awareness	-0.063	0.577	Negative
	Social communication	-0.146	0.210	Negative
	SRS-2 total scores	-0.146	0.207	Negative
	Emotional symptoms	-0.222	0.039	Negative

	SDQ total scores	-0.144	0.217	Negative
Total	Social awareness	0.314		Positive
	Social communication	0.251		Positive
	SRS-2 total scores	0.112		Positive
	Emotional symptoms	-0.198		Negative
	SDQ total scores	0.041		Positive

The “Total” on the right shows the total partial Spearman's correlation coefficients between the altered gut metabolites and autism-related symptoms. Autism-related symptoms include ASD symptom severity, social awareness, social communication, social responsive scale-second edition (SRS-2) total scores, emotional symptoms, and strengths and difficulties questionnaire (SDQ) total scores. Adjusted for child's age, sex, intellectual functioning, advanced maternal age, feeding patterns before 2 years of age, daily sleep duration, daily moderate-to-vigorous physical activity (MVPA) duration, and parenting behavior. ASD, autism spectrum disorder.

Supplementary Table 5. The correlation between constipation and altered gut metabolites in children with ASD (n = 90).

Variables	Altered gut metabolites	Correlation coefficients	P-value	Negative_Positive
Constipation	Chenodeoxycholic acid	0.252	0.016	Positive
Constipation	Arachidonic acid	-0.235	0.025	Negative
Constipation	Palmitic acid	0.236	0.025	Positive
Constipation	Glutaric Acid	0.310	0.003	Positive
Constipation	Choline	-0.395	<0.001	Negative
Constipation	Total	0.168		Positive

Constipation was evaluated by subscale scores of the 6-item gastrointestinal severity index (6-GSI). The "Total" on the right shows the total Spearman's correlation coefficients between constipation and the five altered gut metabolites. ASD, autism spectrum disorder.

Supplementary Table 6. The partial correlation between constipation and altered gut metabolites in children with ASD (n = 90).

Variables	Altered gut metabolites	Partial correlation coefficients	P-value	Negative_Positive
Constipation	Chenodeoxycholic acid	0.281	0.011	Positive
Constipation	Arachidonic acid	-0.246	0.027	Negative
Constipation	Palmitic acid	0.262	0.018	Positive
Constipation	Glutaric Acid	0.343	0.002	Positive
Constipation	Choline	-0.407	<0.001	Negative
Constipation	Total	0.233		Positive

Constipation was evaluated by subscale scores of the 6-item gastrointestinal severity index (6-GSI). The "Total" indicates the total partial Spearman's correlation coefficients between constipation and the five altered gut metabolites. Adjusted for child's age, sex, intellectual functioning, advanced maternal age, feeding patterns before 2 years of age, daily sleep duration, daily moderate-to-vigorous physical activity (MVPA) duration, and parenting behavior. ASD, autism spectrum disorder.
